# How human practices have affected vector-borne diseases in the past: a study of malaria transmission in Alpine valleys

**DOI:** 10.1186/1475-2875-6-115

**Published:** 2007-08-29

**Authors:** Julien Sérandour, Jacky Girel, Sebastien Boyer, Patrick Ravanel, Guy Lemperière, Muriel Raveton

**Affiliations:** 1 Laboratoire Ecologie Alpine, UMR CNRS N°5553, Equipe Pertubations Environnementales et Xénobiotiques, Université Joseph Fourier, BP 53X, 38041 Grenoble Cedex 09, France

## Abstract

**Background:**

Malaria was endemic in the Rhône-Alpes area of eastern France in the 19^th ^century and life expectancy was particularly shortened in Alpine valleys. This study was designed to determine how the disease affected people in the area and to identify the factors influencing malaria transmission.

**Methods:**

Demographic data of the 19^th ^century were collected from death registers of eight villages of the flood-plain of the river Isère. Correlations were performed between these demographic data and reconstructed meteorological data. Archive documents from medical practitioners gave information on symptoms of ill people. Engineer reports provided information on the hydraulic project developments in the Isère valley.

**Results:**

Description of fevers was highly suggestive of endemic malaria transmission in the parishes neighbouring the river Isère. The current status of anopheline mosquitoes in the area supports this hypothesis. Mean temperature and precipitation were poorly correlated with demographic data, whereas the chronology of hydrological events correlated with fluctuations in death rates in the parishes.

**Conclusion:**

Nowadays, most of the river development projects involve the creation of wet areas, enabling controlled flooding events. Flood-flow risk and the re-emergence of vector-borne diseases would probably be influenced by the climate change. The message is not to forget that human disturbance of any functioning hydrosystem has often been linked to malaria transmission in the past.

## Background

Malaria remains a major global public health problem, responsible for the death of one million people each year, and threatening more than three billion people in 107 countries [1]. This ancient disease is transmitted to people by anopheline mosquitoes and has been controlled in many countries, and eradicated in some.

The disease spread widely since the 16^th ^century, from the Mediterranean basin up to Britain [2,3], Denmark and Sweden [4,5]. In France, a study carried out by the Société Royale de Médecine in the surroundings of Paris reported more than 1,400 cases of fever per month from 1783 up to 1785 [6]. In the late 19^th ^century, the area of Les Dombes was particularly affected by malaria; the average life expectancy was 24 years (as compared with 35 for the French population overall) and up to 94% of the inhabitants were infected [7]. Until the beginning of the 20^th ^century and the  discovery by Ross and Grassi of malaria transmission by *Anopheles *mosquitoes in 1903, the control of this disease was inefficient and disorganized.

Despite the severity of the climate during the Little Ice Age (15^th^–19^th ^century), malaria was still transmitted by *Anopheles maculipennis *s.l. mosquitoes, which survived by over-wintering in human habitations and cattle shelters [5,8]. It is, therefore, assumed that *Plasmodium vivax *was the major malaria pathogen in Europe until its official eradication in 1975, judging from the symptoms described in humans infected by the parasites (tertian fever) and in view of its ability to survive cold winters. However, *P. vivax *and *Plasmodium falciparum *may have both been involved [9]. In fact, *P. falciparum *was present in the 1930s from Southern Europe [6,10] to the northern Soviet Union [4]. The *P. vivax *vector, *Anopheles atroparvus*, may also have been able to transmit *P. falciparum*, in southern Europe [11].

Changes in land use and the programme launched by the World Health Organization (WHO) at the end of the 1950s succeeded in eradicating malaria in most of the countries of the WHO European region, except for Turkey, Azerbaijan and Tajikistan, where residual foci of malaria remained intact [12]. The last outbreak in metropolitan France was reported from Corsica in the 1970s [13]. Nowadays, all the malaria cases are imported from endemic malaria areas, except for recent suspected autochthonous cases reported in Corsica [14,15].

In recent years, numerous authors have stated that vector-borne diseases such as malaria would probably spread further at their northern limit with anticipated climate change [16,17]. In this context, epidemiologists have tried to evaluate the risk of autochthonous malaria in Western Europe once more [8,18,19]. They all concluded that the risk is low, but encouraged assessments of risk factors for an outbreak in malaria-free territories. In this article, the causes of endemic malaria transmission in Alpine valleys in the 19^th ^century were studied. The relationships between meteorological conditions, hydraulic operations and malaria cases was investigated using several archive sources on local flooding management, fever symptoms and mortality data. The malariogenic potential of this territory is investigated and the risk factors for malaria resurgence in the context of global change are discussed.

## Methods

**Figure 1:**
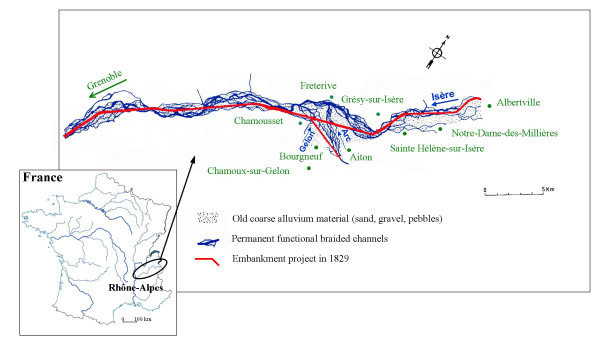
Map of the Isère Valley in Rhône-Alpes before river embankment.

Historical investigations were carried out in order to explain the factors influencing malaria transmission in the region of the Isère (France) in the 19^th ^century. Information on the evolution of mortality in the 19^th ^century in villages of the alluvial flood-plain in the surroundings of Aiton (Figure 1) was collected from death registers. The villages of Notre-Dame-des-Millières (1,030 inhabitants in 1844), Sainte-Hélène-sur-Isère (population not documented, n.d.), Grésy-sur-Isère (n.d.), Aiton (890), Fréterive (n.d.), Bourgneuf (406), Chamousset (301) and Chamoux-sur-Gelon (1,409) were chosen for their situation in a wide area of marshy land. The number of deaths per year was calculated for each village from death registers at the town hall. Data was available as a listing of deaths recorded during a ten year period, starting in 1828, except for the villages of Chamousset and Sainte-Hélene-sur-Isère, where some data were missing.

As the cause of death was not indicated in death registers, medical data reported from physicians and doctors in the district archives provided information on the transmission of fevers and on the symptoms in the population. Description of these symptoms were of good quality as doctors suspected marsh fevers to be responsible for observed mortality peaks in the locality. The following words used for the disease are highly suggestive of malaria infection: "fièvres paludéennes", "fièvres intermittentes" (intermittent fever), "fièvres tierces" (tertian fever). Archive reports from engineers were informative on the causes and the chronology of flood management operations occurring in the Isère valley. All these data were collected from the Archives Départementales de la Savoie, Chambéry, France ("Archives administratives de la période sarde (1816–1860): série FS: génie civil et travaux publics"; "Travaux publics depuis 1860 (série S): Fonds du service des Ponts-et-Chaussées, sous-série SPC").

Meteorological data between 1800 and 1900 were available from the European Alps Temperature and Precipitation Reconstructions data set [20]. Monthly estimated temperature and precipitation for the Albertville surroundings (45.25°N – 6.25°E) were analysed in order to estimate the influence of weather conditions on local mortality records. Correlation was investigated between death records in an area and temperature or precipitation data for the same period with the StatView 4.57.0.0 software.

## Results

### Evolution of mortality in parishes bordering the Isère river

Calculations of the mortality rate in some villages surrounding the river Isère gave evidence of a rapid and significant increase in deaths in stable populations between 1828 and 1843 (Table 1). This increase in mortality rate (+ 21% in Aiton to + 167% in Notre-Dame-des-Millières for 15 years) was not observed in the population of the rest of France for the same period (from 2.51 to 2.35: – 6%) [21,22], suggesting that the phenomenon was geographically localized.

**Table 1:** Comparison of mortality rates in villages bordering Isère river between 1828 and 1843. Mortality rate for Alpine villages were calculated with data from [25,26]

**Villages**	**Mortality rate % (1828–1836)**	**Mortality rate % (1837–1843)**
Aiton	4.86	5.86
Bourgneuf	3.66	5.63
Chamousset	3.32	5.32
Chamoux-sur-Gelon	2.67	3.86
Notre-Dame-des-Millières	2.78	7.44

At this time, local doctors explained this increase in mortality in the valley by a bad flow of the streams in the flood-plain, due to a neglect of maintenance of hydraulic developments [25]. Two reasons were advanced for the increase of marsh fever transmission in the population: first, the embankment operation of the river Arc resulted in the raising of the water level, not suitable for the discharge of the tributaries; and second, the artificial silting of the waterholes on the sides of the dike was not undertaken.

Registered data from the psychiatric hospital of Betton, in the vicinity of Aiton, provided important testimony of fever progression in the locality for this period [26]. The hospital was opened in 1828 in a place reported as free from marsh fever by a medical investigation. In 1834, the medical staff of Betton hospital noticed that residents and employees were suffering from intermittent fever. The unhealthiness of the area was explained by the foul discharge from the local river Gelon (Figure 1), due to previous flooding management of local streams. In 1842, the hospital doctor reported that, despite the abundant prescription of quinine, 20 of the 135 patients died affected by fever in the same year.

These reports and the description of fever are consistent with the likelihood of malaria infections both in hospital residents and in the local population. Malaria transmission by *Anopheles *mosquitoes was, therefore, probably an additional factor causing the death of sick people in the area.

### Anopheline populations in Alpine valleys

Anopheline populations of Alpine valleys were probably not monitored in the 19^th ^century, as malariologists did not consider them to be involved in malaria transmission before  the work of Ross (1897) and Grassi (1898). Alpine *Anopheles *were first studied in the 20^th ^century. There are 13 French Anopheline species [27] and most of them are not considered to be anthropophilic, except *Anopheles labranchiae*, *Anopheles superpictus *and *Anopheles sacharovi*, which are only found in Corsica. These species are known to have been primary vectors of *P. vivax *malaria in Mediterranean countries. This suggests that Corsica had malariogenic potential [28]. A recent study in the Camargue reports the anthropophilic behaviour and spectacular aggressivity of *Anopheles hircanus*, which is abundant in southern France, but not observed in Alpine valleys [15]. The species *An. atroparvus *feeds on mammals and humans. It was implicated in the transmission of malaria in continental France until 1943 and was still responsible for nuisance in Alpine valleys in the 1980s [29,30]. Studies showed that adults were not successfully infected by older European strains of *P. falciparum*before its eradication [69], but that they were sensitive to *P. vivax *[31-33]. No relevant density was observed and the species seemed to have been progressively replaced by zoophilic *Anopheles messeae *in the Rhône-Alpes area since the end of the 19^th ^century [34]. Species considered as occasional malaria vectors (i.e. *Anopheles claviger, An. messeae *and *Anopheles plumbeus*) are currently found in the area. Autochthonous cases of malaria transmission have been attributed to *An. plumbeus *in England and Germany [35,36] and infectivity experiments have confirmed the sensitivity of European *An. plumbeus *to African strains of *P. falciparum*. This species was responsible for nuisance every summer in some towns of the Rhône-Alpes region (D. Rey, Entente Interdépartementale pour la Démoustication, personal communication).*Anopheles messeae *and *An. claviger *are considered to be zoophilic but could have been implicated as secondary vectors in malaria transmission.

There is no doubt that malaria vectors were present in Alpine valleys in the 19^th ^century and fluctuations in their density and longevity probably explained the pattern of mortality rates in villages. As breeding site suitability and mosquito development are influenced by temperature and humidity, relations between meteorological and mortality fluctuations were analysed.

### Weather conditions and mortality

**Figure 2:**
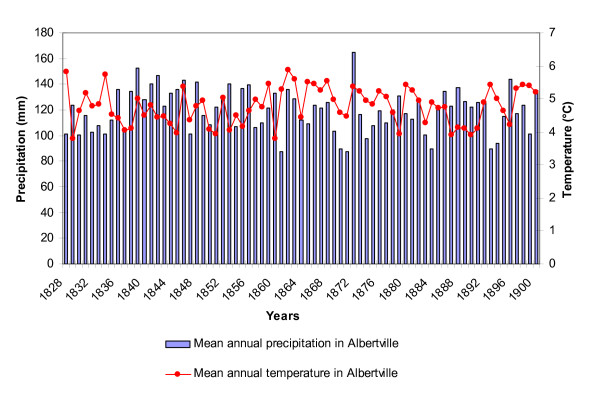
Mean annual precipitation and temperature in Albertville between 1828 and 1900. Adapted from [22].

Alpine valleys have an unusual set of topographic and meteorological conditions that could be linked to local increases in mosquito population density. Analysis of temperature fluctuations for the Albertville area between 1800 and 1900 showed no major climatic event (Figure 2). However, temperatures in June seemed correlated with observed mortality in the following year (R^2 ^= 0.102; Anova F = 8.06, p = 0.0059, 1 df). An analysis of variation in precipitation for the same area showed that neither annual nor monthly precipitation showed correlation with mortality data (Figure 2; P > 0.05).

Weather conditions probably influenced *Anopheles *biology and could be an explanation for the high numbers of malaria cases. However, the importance of other factors potentially involved in mosquito habitat development and mosquito density increases were analysed.

### Flood management operations of the river Isère and mortality

**Figure 3:**
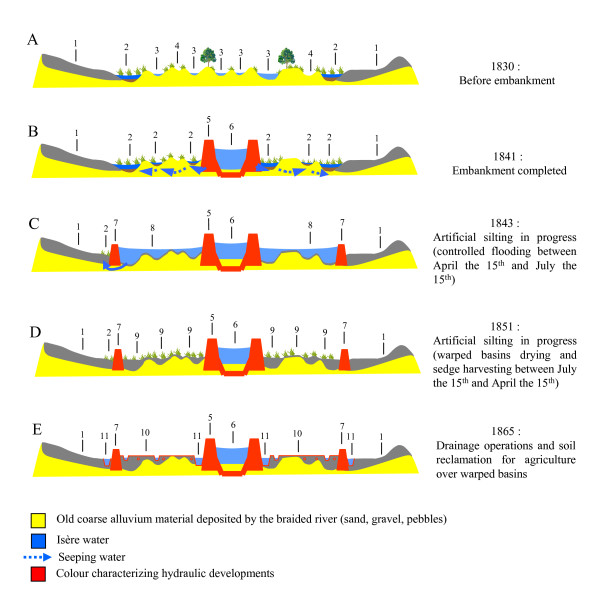
Schematic cross-section in the upper Isère river valley (upstream from confluence with Arc river) illustrating the evolution of hydraulic operations (period 1830–1865). 1: Farmed silty soils on old alluvial terraces. 2: Permanent mosquito-breeding habitats (old river channels flooded by seeping water). 3: Permanent functional braided channels. 4: Braided channels flooded during high-flows periods. 5: Embanked/channelized river. 6: Man-made canal (new channel) more or less filled by alluvial deposits. 7: Berm delineating warped basins. 8: Warped basin flooded by a layer of flowing water. 9: Warped basin more or less filled up by fine alluvial deposits (temporary mosquito breeding habitats on waterlogged soils). 10: Reclaimed areas for farming after drainage (ditches) and underdraining operations (tiles). 11: Lateral canal conveying seeping water and small tributaries to downstream.

**Figure 4:**
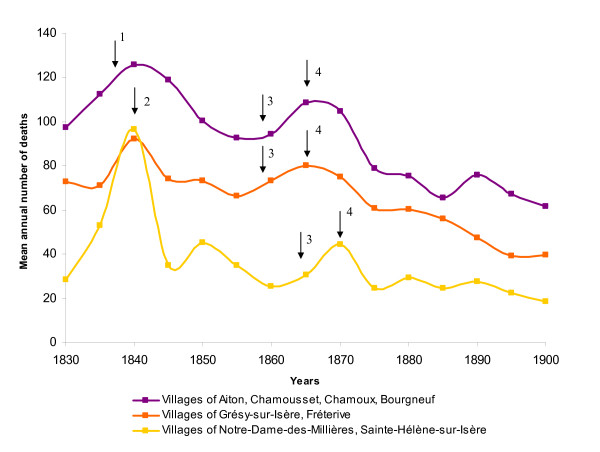
Evolution of mortality between 1830 and 1900 in villages situated in the vicinity of the confluence between Arc and Isère rivers. 1: Recurrent flooding of Arc and Gelon rivers consecutive to channelization. 2: Embankment of Isère river completed. 3: Artificial silting in progress. 4: Drainage operations were beginning.

At the beginning of the 19th century,  Isère river was a braided system  consisting in numerous channels and associated swamps which were famous foci of  marsh fever [37,38]. During the summer, flood-level decrease favoured the proliferation of temporary ponds and the local population lived on harvesting aquatic vegetation for cattle breeding. Stagnant water was considered as the main cause of unhealthiness and fevers. In 1829, the Sardinian government decided to reduce local morbidity in the flood-plain by building embankment by the river (Figure 1). Engineers' archives record the different steps in the hydraulic development of the river (Figure 3) and the local evolution of fever cases. From 1830 to 1854, the embankment work on the river took place. The aim of this work was to drain the neighbouring marshes (Figure 3A). Many workers coming from the neighbouring villages were infected by marsh fever [39] and mortality in the parish increased (three-fold higher in 1840 than in 1830, Figure 4). Despite the dike, the river branches remained connected by dripping waters and infiltration during spring floods, favouring the increase of wetland surface [37] (Figure 3B). In the vicinity of Grésy-sur-Isère, the river flowed at a higher elevation in its artificial channel as compared to its natural level which resulted in recurrent floods during high-flow periods (from mid-April to mid-July) and creation of temporary pools. The architects decided to fill up the marshes and pools by way of an artificial  silting operation called "warping" ; part of the river flow was diverted into  specially-constructed basins in which sediment deposited during the floods. The  clear water then returned to the river further downstream. (Figure 3C). Aquatic macrophytes rapidly colonized these plunge pools and valuable vegetative biomass was harvested during the summer (Figure 3D); the following years, fever cases and mortality increased once more (+75%, Figure 4) [24]. New policies adopted in 1865 resulted in better cooperation between neighbouring villages permitting an efficient circulation of water along the drainage basin and the complete silting of marshes (Figure 3E). These hydraulic works contributed to the decrease of mortality (-60% between 1870 and 1900) and fever cases in the Isère valley (Figure 4) [40]. The correlation of dates between hydraulic operations and mortality events in the parishes of Notre-Dame-des-Millières and Grésy-sur-Isère provide evidence that water management was the cause of fever progression.

### Flood management operations along the river Arc and mortality

The new channel of the river Arc (Figure 1) was built from 1830 to 1839, short-cutting the natural braided river. As a result, from 1832, the progressive raising of the channel bed by the accumulation of sediment downstream from this river was responsible for recurrent flooding of the river and its tributary, the river Gelon, over a farmed area [38]. The consequence was an increase in fever cases at Bourgneuf, Aiton and Chamoux and recorded mortality started to increase from 1830–32 onwards, with a peak of 126 annual deaths between 1840 and 1845 (Figure 4).

The progressive diversion of the river Gelon into the river Isère from 1842 to 1854 limited floods and mortality decreased from 1842 to 1845 with a minimum of 93 deaths between 1855–60. Artificial silting works in the Arc and Isère valleys took place at the same time, favouring the creation and persistence of marshy lands in the area (more than 1000 ha along the river Gelon, Figure 3D). Consequently, the observed fever cases and the number of deaths increased significantly with a new peak between 1865 and 1870 (Figure 4). Drainage operations in the valley definitely removed marshy lands by 1880, as seen previously with the river Isère (Figure 3E) [38,41], and a significant decrease in the number of sick people and deaths was recorded in the vicinity of the river Gelon and the river Arc (Figure 4).

Channelizing rivers, artificial silting operations and drainage network development were disturbance patterns, and played a major role in water-table and flood-level fluctuations over space and time [42,43]. Fluctuation of mortality rates in the parishes located around the confluence seem closely linked to the variation of hydrological factors.

## Discussion

### Vector density was the link between flood management and mortality fluctuations

Mean temperature recorded in June between 1828 and 1900 were correlated with the number of deaths the following year. In Finland, similar correlations were observed between summer temperature and malaria deaths the following year for the same period [5]. The authors explained that summer temperature probably regulated the density of vectors in adult stages. In alpine valleys, *Anopheles *were currently observed in the larval stages in spring. Thus fluctuations in June temperature probably influenced the capacity for larvae to reach the adult stages and therefore vector density and malaria transmission. Nevertheless, weather conditions did not explain *Anopheles *density in larval stages and other factors related with hydrosystems functioning were probably implicated in the high mortality rates.

As demonstrated in this study, mortality rates in the Isère valley in the 19^th ^century were linked to the river-water alluvial flood-plain management. Demographic data were consistent with periods of expansion and decrease of mosquito habitats, suggesting that malarial fevers transmitted by *Anopheles *were related to local death rates. Agricultural practices in the flood-plain consisted of harvesting sedge for cattle during summer, when adult mosquito densities were at their highest.

Before flood management was introduced, secondary arms of the braided rivers dried up in summer, favouring the proliferation of temporary ponds suitable for mosquito breeding. Outdoor contact between people and adult *Anopheles *occurred in late summer and in autumn. After the embankment of the river Isère and its tributaries, breeding sites for *Anopheles *were created by infiltration and overflow during the spring flood. The stagnation of water in the valley created conditions suitable for mosquito proliferation. Consequently, the high *Anopheles *population densities must have increased malaria transmission during summer in the marshy valley and must explain increasing mortality in the parish. During the first years of silting operations in the flood-plain, the presence of valuable vegetation and residual ponds colonized by *Anopheles *in the warping basins from mid-summer onwards, facilitated contact between vectors and people during late summer. This prolonged contact between humans and mosquitoes was probably favourable to the outdoor transmission of malaria until autumn. As dyking works on the river Isère were carried out progressively from downstream (Grésy-sur-Isère) to upstream (Notre-Dame-des-Millières), artificial silting-up began later upstream. This time-lag between hydraulic operations could explain the delay in mortality peaks observed in the two parishes in 1860–70. The complete silting-up of the flood-plain and its drainage may have progressively eliminated ponds suitable for *Anopheles *development, and could explain the definitive decrease of mortality rate in local populations since 1880.

During the period of endemic malaria, an indoor transmission of *Plasmodium *was also suspected in winter, as reported in other countries [9,5]. Indeed, *An. atroparvus*, which is considered to be the primary vector of malaria in France, could be found in farm buildings and it remained semi-active and occasionally feeding during winter [44-46].

The more dangerous species of *Plasmodium*, *P. falciparum*, was present for a long time in southern Europe in the 19^th ^century [10]. However, its implication in malaria epidemics in alpine valleys may have been outclassed by *P. vivax*, which is more robust in the face of the hard climatic conditions that occurred in winter in this place. Even if *P. vivax *has nowhere been reported as a direct cause of death, recent studies have demonstrated that its burden was underestimated and that it should no longer be considered responsible of benign malaria [47,48]. In alpine valleys like in the English marshes, vivax malaria must have been one of the determinants of unhealthiness, in interaction with poor nutrition and other diseases [49] even if epidemics like cholera in 1854 and 1867 had not a serious impact on demography (less than 1% of the Savoie population died from cholera in both epidemics, which were restricted to the main towns; [50]).

The availability of quinine derivatives for anti-malarial treatments certainly had a significant impact in malaria decrease in the end of the 19th century in Europe [4,51]. In the Rhône-alpes region, the use of quinine is poorly documented, and it was probable that most of rural people could not buy it [26]. However, the decline in quantities of quinine sold by an office of the Isère valley in 1867 suggested that malaria cases were decreasing [52], which was concordant with mortality data of the period.

### Environmental management related to malaria transmission

The case study of malaria transmission in Alpine valleys is not unique. Historically, epidemic marsh fevers have often been associated with poor water management. Consequently, regional measures have sometimes been taken to control morbidity. In Italy, Toricelli (1665) recommended the silting-up of alluvial marshlands by encouraging storage and deposition of flood sediments in the marshes [53,54]. In the surroundings of Pisa and Lucca, in the 18^th ^century, the drainage of freshwater and seawater was followed by a rapid decrease in malarial fever cases in the area [55].

In the Rhône-Alpes region of France, at the end of the 18^th ^century, a drainage network in the flood-plain of the river Leysse was established in order to irrigate meadows and harvest vegetation. Recurrent flooding of the river favoured mosquito breeding-site establishment and led to the destruction of the irrigation system. Consequently, malaria cases increased drastically around 1830, with 50% of the parish population being ill, as *Anopheles *benefited from stagnant water in the neglected drainage network [56]. Malaria was eradicated after the dyking of the river Leysse, efficient drainage and reclamation of the flood-plain (1850–1870). In the same way, the vast operation of drainage (almost 100 000 ha) of Pontine marshes (Italy) undertaken in the 1930s by Mussolini succeeded to the previous failed attempt during the Roman period and improved the global healthiness of the parish [4,57].

In most cases, a decrease in malaria was followed by a period of further and more serious outbreak of the disease due to carelessness in the maintenance of equipment and hydraulic developments. Moreover, changes in agricultural practices, urbanization and the building of transport networks led to a considerable alteration in the function of the hydrosystem and were also factors implicated in new malaria outbreaks [10,58,59].

### Perspectives in a changing environment

Progress in human health and hygiene associated with the availability of quinine derivatives for anti-malarial treatments may have had a significant impact in malaria decrease in the early 20^th ^century [60-62]. Life style has progressively changed during the 20^th ^century, with the desertion of many rural areas, the modification of agricultural practices and land use [4,63]. Since the end of the 19^th ^century numerous wetlands have disappeared, disconnected from waterways or drained for agriculture and urbanization development needs. At the present time the most important breeding sites for mosquitoes in the Rhône-Alpes area are located in flood-plains of the river Rhone and its tributaries. Currently, Alpine valleys have no malariogenic potential due to a low density of anthropophilic *Anopheles *populations.

Today, scientists agree about the existence of global warming and that vector-borne diseases could spread further at their northern limits with climate change [17]. Mosquitoes and *Plasmodium *species are highly sensitive to climatic factors; moisture and temperature influence their development and survival [8,16]. Therefore, progressive warming would theoretically influence the vectorial capacity of mosquitoes and, subsequently, malaria transmission, if they are infective [64]. Currently, ancient primary vectors like *An. labranchiae *and *An. superpictus *have density relevant for malaria transmission in European countries [18]. However, this situation of anophelism without malaria tends to show that the vulnerability of western Europe, in the global warming context, is low due to the quality of socio-economic and environmental conditions [51].

Mountains and in particular the Alps are susceptible to the effects of rapid climate change [65]. Glacial ice melting and fluvial flooding will increase and predictions are that flood flows may increase by as much as 20% by 2050 [63,66]. Preventative hydraulic operations are, therefore, planned, for example in the valley of the Isère, where the last serious flood occurred in 1859. According to the Integrated Upstream Isère Project, agricultural lands upstream of Grenoble will be used to convey and store flood-waters of the river Isère in a few years [67]. The river bed will be dredged in order to increase its water-carrying capacity and the project includes the restoration of riverside ecosystems like wetlands. This kind of development project, based on the return of river sections to their natural state, may favour the creation of temporary ponds and pools suitable for a colonization by mosquitoes. Alpine anopheline populations could, therefore, develop in the future, justifying a preventative investigation of factors suitable for malaria outbreaks. As these flood management strategies are relatively recent [68], only speculations can be made on what future hydrologic landscapes may be, and what it will change for these areas in terms of malaria vulnerability, in a global change context. If historical studies provide explanations for past epidemics, they could also highlight future health policy challenges.

## Conclusion

As reported in this study, malaria transmission in the past has often been linked with the functioning of hydrosystems in western Europe. Hydraulic developments have had a direct influence on the evolution of the fever cases in marsh parishes of Alpine valleys. Today, the flood-flow risks associated with climate-change forecasts are increasing and thus river management strategies are modified. Most of the recent river development projects plan to let the river recover its old and natural way, favouring the creation of temporary reservoirs and wetlands. Even if conditions are not met for malaria resurgence and maintenance in Alpine valleys nowadays, past situation must be considered in order to prevent the occurrence of suitable conditions for both vectors and pathogens.

## Authors' contributions

JS and JG contributed equally to this study, in conceiving its design, collecting and analysing the historical data, and in writing of the manuscript. SB and PR helped to draft the manuscript. GL and MR participated in the design of the study, they supervised the data analysis and helped to draft the manuscript.
